# The Judgment of the CJEU of 25 July 2018 on Mutagenesis: Interpretation and Interim Legislative Proposal

**DOI:** 10.3389/fpls.2019.01813

**Published:** 2020-03-03

**Authors:** Juan Antonio Vives-Vallés, Cécile Collonnier

**Affiliations:** ^1^ University of the Balearic Islands, Palma de Mallorca, Spain; ^2^ Community Plant Variety Office, Angers, France

**Keywords:** mutagenesis, gene editing, GMO, Directive 2001/18/EC, C-528/16, Court of Justice of the European Union, plant breeding, plant biotechnology

## Abstract

The Judgment of 25 July 2018 of the Court of Justice of the European Union (CJEU)^1^ was optimistically awaited by breeders and supporters of agricultural biotechnology, but shortly after the press release advancing the Judgment, hope turned into frustration. Opinions on how to frame the New Breeding Techniques (NBT) in the context of Directive 2001/18/EC were issued before the Judgment, while proposals to assist the EU legislator to amend the regime driven by the Directive have been also provided afterwards by scientists and institutional bodies around the EU. However, they do not seem to have paid so much attention to the Judgment itself. This paper focuses on the Judgment. It finds out that while the impacts of the Judgment on the NBT might have been slightly overvalued, its potential negative effects on techniques of random mutagenesis and varieties breed through them have been generally underestimated if not absolutely overlooked. The analysis also shows that the Judgment does not preempt the possibility to exempt certain applications of some NBT from the scope of Directive 2001/18/EC,^2^ and, in fact, ODM, SDN1, and SDN2 might be, under certain conditions, easily exempted from its scope without the need of a deep legislative revolution nor even the amendment of Directive 2001/18/EC. As regards techniques of random mutagenesis and mutant varieties bred by means of those techniques, until action is taken by Member States (if finally taken), no real limitations upon them are to be feared. However, if Member States start to consider the path opened by the CJEU, then their regulation at an EU level should be readily explored in order to avoid further negative effects on plant breeding as well as on the free movement inside the EU of those varieties and the products thereof.

## Introduction

After a fairly pro-biotech Opinion of Advocate General Bobek^3^ [see, e.g., Purnhagen et al. (2018a; 2018b) and Callaway (2018)], the Judgment of 25 July 2018, *Confédération paysanne and Others*, C-528/16, EU:C:2018:583 of the Court of Justice of the European Union (CJEU) (from now on “the Judgment” or “*Confédération paysanne and Others*”) deeply disappointed the scientific community [see, e.g., Callaway (2018) or Urnov et al. (2018)], because it “classifies genome-edited plants as genetically modified organisms (GMOs) and thus subjects them to prohibitive premarket risk evaluations” (Urnov et al., 2018: 800). After the Judgment, scientists [see, e.g., Urnov et al. (2018)], advisory bodies, such as the German Bioeconomy Council [see Bioökonmierat (2018)] and the European Commission's Group of Chief Scientific Advisors [see SAM (2018)], and more recently even the European Commissioner for Health and Food Safety (Michalopoulos, 2019) have urged to review Directive 2001/18/EC in order to overcome the Judgment. However, a deeper analysis of the Judgment and its impact on the EU legal regime on GMO seems to be needed. This paper focuses on the Judgment, aiming to debunk some myths around it and clarify its meaning, and to present a proposal addressed to mitigate its potential negative effects on plant breeding, the EU legal regime on GMO and the internal market.

## Materials and Methods

The **paper is organized in three sections. The section *Interpretation of the Judgment of the CJEU of 25 July 2018* focuses on the interpretation of the Judgment; the section *Leeway to Operate Out of the Scope of Directive 2001/18/EC Post Judgment* analyzes the leeway to operate out of the scope of Directive 2001/18/EC post Judgment by means of a legislative proposal expressly designed with that purpose; and the section *Analysis of the Impact of the Judgment on the Breeding Techniques* focuses on the impact of the Judgment on the legal status of breeding techniques, and assesses the feasibility and potential usefulness of the legislative proposal outlined in the section *Leeway to Operate Out of the Scope of Directive 2001/18/EC Post Judgment*.

In the section *Interpretation of the Judgment of the CJEU of 25 July 2018*, the Judgment is analyzed by means of the application of well-known principles and rules of legal interpretation in the EU and taking leverage on the analysis of the relevant literature on the EU legal regime on GMO [most remarkably Spranger (2015) and Krämer (2015)]. This exercise has been enrichened with the analysis of relevant reactions to the Judgment from the industry, politicians, and scientific scholars (gathered from websites, electronic newspapers, papers, and other electronic sources found through searches on Google, Google Scholar, WoS, or from pgrip.org). Recourse has also been made to the Opinion of Advocate General Bobek and the Decision^4^ of the referring French court.

In the section *Leeway to Operate Out of the Scope of Directive 2001/18/EC Post Judgment*, trough legal reasoning and on the basis of the analysis performed in the section *Interpretation of the Judgment of the CJEU of 25 July 2018*, a prospective exercise on the leeway to operate out of the scope of Directive 2001/18/EC post Judgment has been conducted, and a legislative proposal has been outlined.

In the section *Analysis of the Impact of the Judgment on the Breeding Techniques*, legal interpretation is combined with scientific/technical analysis to assess the impact of the Judgment on the legal status of the existent breeding techniques (on the basis of the analysis performed in the section *Interpretation of the Judgment of the CJEU of 25 July 2018* and a nonexhaustive literature review on breeding techniques). The possibility to exempt the breeding techniques assessed in this section by means of the legislative proposal outlined in the section *Leeway to Operate Out of the Scope of Directive 2001/18/EC Post Judgment* has been also evaluated.

## Results and Discussion

### Interpretation of the Judgment of the CJEU of 25 July 2018

#### A “Shocking” Decision

After the press release of the CJEU on its Judgment, “shocked” (Michalopoulos, 2018; Science Media Centre, 2018) and “disappointing” (Callaway, 2018: 16; Science Media Centre, 2018) were likely the words best summarizing the mood of the industry and scientists. Since then, beyond some scarce exceptions in which scholars have shown a greater awareness toward the difficult task of the Court [see, e.g., Leyser (2018) and Purnhagen et al. (2018a)], the Judgment and the EU legal regime on GMO have been the target of a general criticism, not only from the Academia and the industry [see, e.g., Michalopoulos (2018) and Urnov et al. (2018)] but also from within EU institutions [see SAM (2018) and Michalopoulos (2019)]. Indeed, after the positive expectations created by the Opinion of Advocate General Bobek (Callaway, 2018; Michalopoulos, 2018; Purnhagen et al., 2018a; Science Media Centre, 2018; Marks and Livingstone, 2019), the Judgment does not bring good news to plant breeders and the agricultural sector (Michalopoulos, 2018; Purnhagen et al., 2018a; Science Media Centre, 2018; Urnov et al., 2018). However, it needs to be noted that the approach of the Court toward NBT, aligned with “the Applicants [Confédération paysanne and Others] together with the French Government” (Opinion of Advocate General Bobek, para 87), is coherent with the European understanding of the precautionary principle in this field as well as with recital 17 Directive 2001/18/EC [anticipated by legal scholars like Krämer (2015) and Spranger (2015), and noticed also by Purnhagen et al. (2018a) and Eriksson (2018)]. Certainly, the role of recital 17 Directive 2001/18/EC acknowledged by the Court contradicts the approach of the Advocate General to recital 17 Directive 2001/18/EC based on historical interpretation (*cf.* paras 90 ff of the Opinion of the Advocate General with paras 44, 51, 54 and the conclusion of the Judgment). However, historical interpretation is far from being the usual approach of the CJEU (Rösler, 2012; Scholz and Cunha, 2017; Purnhagen et al. 2018a). That art 3(1) Directive 2001/18/EC is to be interpreted “strictly” (para 41 of the Judgment) was already anticipated by Krämer (2015) and Spranger (2015). Besides, the need to take into account “the context [ … ] and the objectives pursued by the rules of which it is part” (Judgment, para 42) and the principle of narrow interpretation of exemptions (Judgment, para 41), are well known rules of legal interpretation in the EU [see, e.g., Scholz (2012a; 2012b) and Beck (2012)] and was also anticipated by Krämer (2015) and Spranger (2015). In fact, the conclusions and reasoning of the Court were anticipated by Krämer (2015) and Spranger (2015) to a large extent. In the light of the foregoing, the Judgment, at least as regards its conclusions on NBT, can hardly be deemed groundless from a legal perspective nor surprising. From a rational and a scientific perspective though, as manifested by some scholars [see, e.g., Leyser (2018) or Purnhagen et al. (2018a)], the Judgment is just as objectionable as the EU legal regime on GMO.

**Directive 2001/18/EC: Recital 17 and art 3(1)**

- Recital 17 Directive 2001/18/EC reads as follows: “(17) This Directive should not apply to organisms obtained through certain techniques of genetic modification which have conventionally been used in a number of applications and have a long safety record.” This recital is connected with the precautionary principle (Krämer, 2015; Spranger, 2015).
- Art 3(1) Directive 2001/18/EC: “This Directive shall not apply to organisms obtained through the techniques of genetic modification listed in Annex I B.”

**The precautionary principle**

The precautionary principle has its roots in the German law (Andrew and O'Riordan, 2004) and its recognized, although not defined, in art 191 of the Treaty of Functioning of the European Union (TFEU) (European Union, 2016), but it has been developed by the Communication from the Commission on the precautionary principle (Brussels, 2.2.2000 COM(2000) 1 final) (Andrew and O'Riordan, 2004). As understood by the Communication from the Commission, “the precautionary principle which enables a rapid response to be given in the face of a possible danger to human, animal or plant health, or to protect the environment. In particular, where scientific data do not permit a complete evaluation of the risk, recourse to this principle may, for example, be used to stop distribution or order withdrawal from the market of products likely to be hazardous” (European Union, 2016).

**Historical interpretation of law**

As explained by Scholz and Cunha (2017): “Historical interpretation, in the case of EU law, relies on the historical background, the content of *travaux preparatoires* [preparatory work] or similar materials, which record the legislators' intention and the purpose for which the provision was made.”

#### Regulatory Changes “On the Way”

After the Judgment, the idea of a revision of the EU legal framework on GMO as a reaction to interpretation of the CJEU [see, e.g., Bioökonmierat (2018); Michalopoulos (2019) and Urnov et al. (2018)] gained momentum, resulting in the “Statement by the Group of Chief Scientific Advisors”^5^ [see SAM (2018)]. However, in the current European context as regards GMO, defined by Directive (EU) 2015/412,^6^ Commission Implementing Decision (EU) 2016/321 of 3 March 2016,^7^ and by the Judgment itself, this headlong rush toward a new GMO legal framework does not seem to have much chances of success in the short term [see also Purnhagen et al. (2018b)]. In fact, hastiness might contribute to further intensify polarization of public opinion in Europe on this issue [see, e.g., in this respect Gelinsky and Hilbeck (2018); Noisette (2019) and Antoniou (2019)] “reducing the possibility of breaking deadlock” (Mandel, 2005: 168) in the short to medium term. Furthermore, it also seems to have unintentionally prevented a sober analysis of some aspects of the Judgment that might have relevant implications on plant breeding and agriculture.

#### The Judgment on “NBT”

The application before the *Conseil d'État* (the French court that referred the questions for preliminary ruling) requested to “revoke Article D. 531-2 of the Environmental Code, transposing Directive 2001/18, which excludes mutagenesis from the definition of techniques giving rise to genetic modification within the meaning of Article L. 531-1 of the code, and ban the cultivation and marketing of herbicide-tolerant rape varieties obtained by mutagenesis” (Judgment, para 20). Therefore, the request was not focused on NBT but on herbicide tolerant crops (Eriksson, 2018; Leyser, 2018) and on mutagenesis (Purnhagen et al., 2018a; 2018b) in a broad sense. Furthermore, as acknowledged by the *Conseil d'État*, “[t]he only herbicide resistant seeds registered in the common catalogue of varieties of agricultural plant species are the result of *in vitro* random mutagenesis. [ … ] no variety of herbicide resistant seed resulting from the directed mutagenesis techniques has yet been included in the common catalogue” (Opinion, para 25). Besides, the Decision of the *Conseil d'État*, the Opinion of Advocate General Bobek, and the Judgment itself have all of them a broader scope than strictly NBT. Even the heading of the press release of the CJEU on the Judgment [see CJEU (2018)] refers to mutagenesis in a broad sense, not only to NBT. In fact, the conclusions of the Court in its Judgment deal more on mutagenesis *lato sensu* and even on the concept of GMO, than on NBT. However, after the Judgment, most of the scientific publications [see, e.g., Callaway (2018) and Purnhagen et al. (2018a)] and press publications [see, e.g., The Irish Times (2019); Marks and Livingstone (2019) or Zimmere (2018)] focused mainly on the impacts of the Judgment on NBT, not on mutagenesis in a broad sense. In the end, the Judgment itself has come to be known as the “ruling on new breeding techniques” [see, e.g., Opoku Gakpo (2018) or Devuyst (2018)]. As shown in the following paragraphs, this bias in the interpretation of the Judgment might be involuntarily concealing some relevant potential effects of the Judgment on plant breeding and free trade in the EU that should be known and, where appropriate, adequately addressed.

#### Varieties Bred Through Traditional Techniques of Random Mutagenesis “Saved From the Purge” of the Court

Due to the shift of attention toward NBT above described, potential implications of the Judgment on traditional techniques of random mutagenesis and varieties thereof have been generally overlooked or misinterpreted [among the very few exceptions see Martin in Science Media Centre (2018), Gelinsky and Hilbeck (2018), CIOPORA (2018), Wanner et al. (2019), or Jorasch (2019)].

The *Conseil d'État*, in its third question referred for a preliminary ruling, asked the CJEU whether “[a]rticles 2 and 3 of [*sic.*] and Annex I B to Directive [2001/18] on the deliberate release into the environment of [GMOs] constitute [ … ] a full harmonization measure prohibiting Member States from subjecting organisms obtained by mutagenesis to all or some of the obligations laid down in the directive or to any other obligation, or do the Member States, when transposing those provisions, have a discretion to define the regime to be applied to organisms obtained by mutagenesis” (para 25 of the Judgment). The Judgment concludes that “[a]rticle 3(1) of Directive 2001/18 [ … ] does not have the effect of denying Member States the option of subjecting such organisms [ … ] to the obligations laid down in that directive or to other obligations” (para 82).

**Directive 2001/18/EC: Recital 17 and art 3(1)**

- Art 2(2) Directive 2001/18/EC: “(2) “genetically modified organism (GMO)” means an organism, with the exception of human beings, in which the genetic material has been altered in a way that does not occur naturally by mating and/or natural recombination;
  Within the terms of this definition:
  - genetic modification occurs at least through the use of the techniques listed in Annex I A, part 1;
  - the techniques listed in Annex I A, part 2, are not considered to result in genetic modification;”.
- Annex I B Directive 2001/18/EC:
  **“TECHNIQUES REFERRED TO IN ARTICLE 3**
  Techniques/methods of genetic modification yielding organisms to be excluded from the Directive, on the condition that they do not involve the use of recombinant nucleic acid molecules or genetically modified organisms other than those produced by one or more of the techniques/methods listed below are:
  1. mutagenesis,
  2. cell fusion (including protoplast fusion) of plant cells of organisms which can exchange genetic material through traditional breeding methods.”

The Court justified its positioning by arguing that “to the extent to which the EU legislature has not regulated those organisms, Member States have the option of defining their legal regime” (para 79), with the only limitation of “compliance with EU law, in particular the rules on the free movement of goods set out in Articles 34 to 36 TFEU” (para 79). This conclusion apparently stems from art 5(3) of the Treaty of the European Union (TEU) and art 2(2) TFEU; i.e., from the principle of subsidiarity and the rules applying to shared competences [see also Purnhagen et al. (2018b)]. Such interpretation has been already well described by the legal literature in relation to the EU legal regime on GMO [see, e.g., Sadeleer (2014) and Weimer (2019)] and was implicitly acknowledged by the Court in previous cases, like *Pioneer Hi Bred Italia*.^8^ However, in *Pioneer Hi Bred Italia* the Court, even if bound by the same legal principles than in *Confédération paysanne and Others*, decides just in the opposite direction. The main reason motivating these diverging decisions seemingly derives from the fact that in *Pioneer Hi Bred Italia* the Court appreciates the “harmonized” (*Pioneer Hi Bred Italia*, para 5) nature of the matter at issue, while in *Confédération paysanne and Others* it does not (see *Confédération paysanne and Others*, para 79: “to the extent to which the EU legislature has not regulated those organisms [ … ]”). Be that as it may, the interpretation of the Court in *Confédération paysanne and Others* was not the only possible reading of Directive 2001/18/EC in relation to mutagenesis.

**Arts 5(3) TEU and 2(2) TFEU**

- Art 5(3) TEU: “Under the principle of subsidiarity, in areas which do not fall within its exclusive competence, the Union shall act only if and in so far as the objectives of the proposed action cannot be sufficiently achieved by the Member States, either at central level or at regional and local level, but can rather, by reason of the scale or effects of the proposed action, be better achieved at Union level.”
- Art 2(2) TFEU: “When the Treaties confer on the Union a competence shared with the Member States in a specific area, the Union and the Member States may legislate and adopt legally binding acts in that area. The Member States shall exercise their competence to the extent that the Union has not exercised its competence. The Member States shall again exercise their competence to the extent that the Union has decided to cease exercising its competence.”

The Advocate General in its Opinion (see paras 115–117) frames two scenarios that in his view might explain the legislative positioning of the EU legislature in relation to mutagenesis: (a) “the EU legislature made a legislative choice. It carried out an evaluation, and on the basis of that evaluation came to the conclusion that all the mutagenesis techniques are to be excluded because they are safe” (Opinion, para 116); or, (b) “by inserting the mutagenesis exemption, the EU legislature did not make any statement about its safety” (para 117). The Advocate General compares the role of the EU legislature in the first scenario with that of “an architect that decided to have a room called ‘mutagenesis' in his house, but who also decided to keep that room empty” (para 116); while in the second scenario, according to the Advocate General, “the architect effectively decided to leave that space called ‘mutagenesis' outside his house” (para 117). The first scenario amounts to full harmonization (see para 116 of the Opinion), while the second scenario would represent a lack of harmonization (see para 117 of the Opinion). Such scheme stems from the legal reasoning formerly mentioned (deriving from arts 5(3) TEU and 2(2) TFEU) on the basis of the equilibrium on which the EU regime on GMO is built [i.e., the compromise between “[t]he protection of human health and the environment” (recital 5 Directive 2001/18/EC) and the principles governing the internal market in the EU (Salvi, 2016)]. However, the Court does not follow any of the options framed by the Advocate General. Instead, the Court decides to square the circle. Indeed, like the Advocate General, the Court is of the opinion that “the EU legislature has not regulated those organisms” (Judgment, para 79), but it assumes a safety assessment of the EU legislature as regards mutagenesis, manifested in recital 17 (see Judgment, paras 44, 45, 51, 54 and conclusion). In other words, the Court interprets art 3(1), Annex I B (1) and recital 17 Directive 2001/18/EC as a minimum threshold of harmonization (Purnhagen et al., 2018b), and therefore, according to the logic set in art 2(2) TFEU: “Member States shall again exercise their competence to the extent that the Union has decided to cease exercising its competence.” Recouping the example of the Advocate General in its Opinion, “the EU legislator would be like an architect that decided to have a room called ‘mutagenesis' in his house” (Opinion, para 116), but that instead of taking the decision “to keep that room empty” [as framed by the Advocate General (Opinion, para 116)], simply left it this way, and therefore, implicitly allowed EU Member States to furnish it (see Judgment, paras 44, 45, 51, 54, 79 and conclusion).

It could be argued that what the Court really has done is to transform an implicit preemption of action of Member States allegedly stemming from recital 17 Directive 2001/18/EC [equivalent to the above mentioned first scenario framed by the Advocate General (Opinion, para 116)], in a renounce of the EU legislature to regulate GMO obtained by means of traditional techniques of random mutagenesis. This interpretation is supported by the very late appearance of a case like *Confédération paysanne and Others*. In other words, if the possibility of traditional mutagenesis being regulated at a national level needed the pronunciation of the Court after so many years, then maybe the scenario of the EU legislature fully harmonizing those techniques should have been seriously considered by the Court as the most plausible option. However, this line of reasoning is somehow countered by the disharmonizing effect of Directive (EU) 2015/412 on the EU legal regime on GMO [see the Opinion of the Advocate General, para 122, Salvi (2016), Purnhagen et al. (2018b) and Wanner et al. (2019)], and, most importantly, this was not the interpretative path taken by the Court.

From the Judgment onwards, three categories of organisms matter in practice: *(1)* non-GMO; *(2)* GMO mentioned in art 3(1) Directive 2001/18/EC obtained through traditional techniques of random mutagenesis; *(3)* GMO falling within art 2(2) Directive 2001/18/EC [among which, according to the Court, organisms obtained by means of NBT are included (Urnov et al., 2018)]. Before the Judgment, varieties obtained by means of traditional techniques of random mutagenesis were claimed to be “an independent third category due to aspects of risk evaluation” (Spranger, 2015: 25), but that view is not clearly recognized in Directive 2001/18/EC, and there was no legal certainty on this issue until the Judgment. As observed by some scholars and breeders' associations [see, eg, Martin in Science Media Centre (2018); Gelinsky and Hilbeck (2018); CIOPORA (2018); Wanner et al. (2019), or Jorasch (2019)], from now on, according to the Judgement, GMO obtained by means of traditional mutagenesis will be able to be subjected by EU Member States “to the obligations laid down in that directive or to other obligations” (para 82 and conclusion 3). The Court is silent on GMO obtained through cell fusion (point (2) of Annex I B Directive 2001/18/EC), but it is foreseeable the interpretation of the Court extends to them.

This “reclassification” done by the Court might have in turn important implications. Indeed, because of the reference of the Court “to the obligations laid down in that directive [Directive 2001/18/EC] or to other obligations” (para 82 and conclusion 3), Member States might regulate the risk assessment of such varieties or its labelling at a national level, or subject them to other conditions and limitations. Furthermore, it cannot be ruled out that, as a result of the Judgment in connection to Directive (EU) 2015/412, the cultivation of such varieties end restricted or even prohibited at a national level in the same way as GM varieties within the scope of Directive 2001/18/EC [on the analysis of Directive (EU) 2015/412 see, e.g., Salvi (2016)]. In fact, in the light of the Judgment, mutant varieties bred through random mutagenesis might end being subjected “to other [national] national obligations” (para 82 and conclusion 3) potentially stricter and more burdensome than those stemming from Directive 2001/18/EC. It is clear from the aforesaid that the decision of the Court, in addition to further disharmonizing GMO regulation in the EU (Wanner et al., 2019), might also result, despite the condition introduced by the Court [of “compliance with EU law, in particular with the rules on the free movement of goods” (para 82)], in obstacles to the free movement of goods. If Member States end eventually following the possibility set by the CJEU as regards traditional mutant varieties, the free movement of goods [see Wanner et al. (2019)], plant innovation and agriculture [see Martin in Science Media Centre (2018)], and consumer choice in the EU, might be severely affected. Therefore, in that case, action should be taken at an EU level to reharmonize this area of the EU legal regime on GMO in order to impede or minimize the aforementioned potential negative impacts.

#### “Euphoria” in the Organic Sector

In a position paper issued before the Judgment, IFOAM stated that techniques falling within the category of “mutagenesis” are not “[a]cceptable for organic breeding” and “[t]o be phased out” (IFOAM Organics International, 2017: 20); and, shortly after the press release of the Judgment [see CJEU (2018)], showed its satisfaction with the position adopted by the Court [see IFOAM EU Group (2018b)]. However, after the Judgment, references to traditional mutagenesis (and to mutagenesis *lato sensu*) practically disappeared from IFOAM's communications [see, e.g., IFOAM EU Group (2018b) and IFOAM EU Group (2019)] despite IFOAM being apparently well aware of the potential reach of the Judgment [see, e.g., IFOAM EU Group (2018a)]. In the light of it, it cannot be excluded that the Court might have gone even further than some players of the organic sector wished. It must not be forgotten that at a worldwide level there are at least 3318 registered varieties obtained through traditional mutagenesis, 55% of them breed before 1990 (IAEA).

#### The Concept of GMO After the Judgment: The Mutagenesis Exemption

In **Table 1**, the concept of GMO under Directive 2001/18/EC as interpreted by the Court is schematized through the criteria that may end with an exemption from the scope of Directive 2001/18/EC. Construed from the legal analysis of the Judgment carried out in this paper, **Table 1** systematizes the interpretative efforts reflected in the literature [New Techniques Working Group (2012); Krämer (2015); Spranger (2015); Krämer (2015); Spranger (2015); Vives-Vallés (2016); Vives-Vallés (2018), Purnhagen et al. (2018a; 2018b), Sprink et al. (2016); Eriksson et al. (2018); Eriksson (2018); Wanner et al. (2019); Custers et al. (2019), etc.] and adapts them either to the situation post Judgment and/or to the purpose of the table. It is worth noting that the reasoning portrayed in **Table 1** is not new, but it was already anticipated by Krämer (2015) and Spranger (2015) to a great extent. Krämer (2015) and Spranger (2015) detected already in 2015 that the key to understand the concept of GMO of Directive 2001/18/EC are not as much the descriptions contained in arts 2(2) and 3, but mainly the logical scheme set in those articles plus their annexes (i.e., whether the lists they refer to/contain are open or closed) interpreted in the light of the precautionary principle and recital 17 Directive 2001/18/EC. Some of these aspects have been also pointed out, before and after the Judgment, by other scholars [see, eg, New Techniques Working Group (2012); Vives-Vallés (2016) or Purnhagen et al. (2018a; 2018b)]. Therefore, according to the Court, how natural those techniques may be is not determinant (Custers et al., 2019) and the techniques listed in Annex I A Part 1 do not exhaust the notion of (nonexempted) GMO of art 2(2)(a) Directive 2001/18/EC. It is instead the inability of those techniques to fit in Annex I A Part 2 and Annex I B Directive 2001/18/EC, that matters the most (see **Table 1**). Certainly, the Court considered in its assessment the definition in art 2(2) Directive 2001/18/EC, mentioning in fact the three requirements it contains [i.e., “alterations made to the genetic material of an organism” (para 28 of the Judgment), “with the exception of human beings” (para 27), and “in a way that does not occur naturally” (para 29)]; but the two first requirements (in paras 27 and 28) are basically prerequirements, and the importance of the third requirement (in para 29) is somehow watered down by the reference of the Court to “the general scheme of that directive [Directive 2001/18/EC]” (para 31) developed in paras 31 to 37 of the Judgment. The consequence of this interpretation is that, as anticipated by Krämer (2015) and Spranger (2015), a technique will lead to a nonexempted GMO (falling within art 2(2)(a) in connection to Annex I A Part 1 Directive 2001/18/EC) if such technique cannot be classified in Annex I A Part 2 nor in Annex I B Directive 2001/18/EC (see **Table 1**). In other words, a dynamic interpretation of the annexes as regards the techniques covered by them is mandatory for Annex I A Part 1 and not possible for the other annexes (Spranger, 2015). It is worth mentioning that, as implicitly acknowledged by the Court (see Judgment, paras 27–38, 40), what is to be understood by “mutagenesis” has nothing to do with the use of “recombinant nucleic acids” (NA) or “genetically modified organisms” (Annex I B Directive 2001/18/EC). Besides, for a GMO produced by means of mutagenesis to be excluded from the scope of Directive 2001/18/EC, in addition to the fulfilment of recital 17, the “condition that they do not involve the use of recombinant nucleic acid molecules or GMOs other than those produced by one or more of the techniques/methods listed in that annex [Annex I B]” (Judgment, para 40) must also be met (see **Table 1**). This was also foreseen by Krämer (2015) and Spranger (2015) and observed after the Judgment by Eriksson (2018). The Judgment however, even if mentioning all these requirements from Annex I B and recital 17, does not elaborate on any of them. Regarding “the condition that they do not involve the use of recombinant nucleic acid molecules” (Annex I B Directive 2001/18/EC), Krämer (2015) explains that Council Directive 90/220/EEC does not contain it and that its inclusion in Directive 2001/18/EC must be understood as “a supplementary requirement [ … ] to enlarge the field of application of Directive 2001/18 and to reduce the exemption of Article 3 and Annex I B” (Krämer, 2015: 12). As regards recital 17, Directive 2001/18/EC does not provide any guidance on its interpretation (Krämer, 2015); but it makes sense to interpret it as containing two different, but cumulative (i.e., both of them must be fulfilled), requirements (Krämer, 2015). Fulfilling “a number of applications” only is not enough [see Krämer (2015)]. Additionally, those applications must “have a long safety record” [see Krämer (2015)]. Directive 2001/18/EC does not explain either how recital 17 must be assessed Krämer (2015). A reading of recital 17 Directive 2001/18/EC coherent with the Judgment [as well as with the position taken by Krämer (2015) and Spranger (2015)] suggests that, somehow, the first requirement in recital 17 (i.e., “a number of applications”) should refer to the diversity of the applications, while the second requirement in recital 17 (i.e., “a long safety record”) might be connected to the number of records within each application as well as to the proven degree of “safety” of each application. It also must be noted that Annex I B refers to “the use of recombinant nucleic acid molecules or genetically modified organisms” but not to “[t]echniques of genetic modification” like Annex I A Part 1; therefore, those techniques of genetic modification that “do not involve the use of recombinant nucleic acid molecules or genetically modified organisms” (as mandated in Annex I B) and fitting also within the scientific notion of “mutagenesis,” should be deemed potentially coverable by Annex I B [see also Purnhagen et al. (2018b)], subjected only to the fulfilment of the requirements in recital 17. Besides, according to the Judgment (see also **Table 1**), the techniques listed in Annex I B must be deemed always included in art 2(2)(a) Directive 2001/18/EC (see Judgment, paras 27–38), and only excluded from the scope of Directive 2001/18/EC as long as they meet the requirements stemming from recital 17 (paras 43–48) and mentioned in Annex I B Directive 2001/18/EC (para 40). The Judgment though, does not provide any guidance on what is to be understood by to “not involve the use of recombinant nucleic acid molecules or genetically modified organisms other than those produced by one or more of the techniques/methods listed” in Annex I B Directive 2001/18/EC; nor on how should the requirements in recital 17 Directive 2001/18/EC be assessed. The term “mutagenesis” is not addressed in the Judgment either.

**Table 1:** Cumulative criteria that genetic engineering/breeding techniques must meet in order to be excluded from the scope of Directive 2001/18/EC as interpreted by the CJEU in the Judgment.

Order/Question	Criteria	Result
1)	Does it result in “an organism [ … ] in which the genetic material has been altered”?	Negative answer: Out of the scope of Directive 2001/18/EC.
		Affirmative answer: Check requirement “2).”
2)	Does it refer to an implementation on “human beings”? (“exception” contained in art 2(2) 2001/18/EC)	Affirmative answer: Out of the scope of Directive 2001/18/EC.
		Negative answer: Check requirement “3).”
3)	Does it fit in any of the techniques listed in Annex I A Part 1 Directive 2001/18/EC?	Affirmative answer: Within the scope of Directive 2001/18/EC (i.e., art 2(2)(a) Directive 2001/18/EC).
		Negative answer: Check requirement “4).”
4)	Does it fit in any of the techniques listed in Annex I A Part 2 Directive 2001/18/EC?	Affirmative answer: Out of the scope of Directive 2001/18/EC.
		Negative answer: Check requirement “5).”
5)	Does it fit in the notion of “mutagenesis” or “cell fusion (including protoplast fusion) of plant cells of organisms which can exchange genetic material through traditional breeding methods”?	Negative answer: Within the scope of Directive 2001/18/EC (i.e., art 2(2)(a) Directive 2001/18/EC).
		Affirmative answer: Check requirement “6).”
6)	Does it “involve the use of recombinant nucleic acid molecules or genetically modified organisms other than those produced by one or more of the techniques/methods listed” in Annex I B Directive 2001/18/EC?	Affirmative answer: Within the scope of Directive 2001/18/EC (i.e., art 2(2)(a) Directive 2001/18/EC).
		Negative answer: Check requirement “7).”
7)	Has it “conventionally been used in a number of applications”?	Negative answer: Within the scope of Directive 2001/18/EC (i.e., art 2(2)(a) Directive 2001/18/EC).
		Affirmative answer: Check requirement “8).”
8)	Has it “a long safety record”?	Negative answer: Within the scope of Directive 2001/18/EC (i.e., art 2(2)(a) Directive 2001/18/EC).
		Affirmative answer: Exempted from the scope of Directive 2001/18/EC (on the basis of art 3(1) Directive 2001/18/EC). It may still be subjected “to the obligations laid down in that directive [Directive 2001/18/EC] or to other obligations” by EU Member States (Judgment, para 82 and conclusion 3).

“Order/Question”: Logical order in which the criteria must be assessed for a given technique. “Criteria”, Criteria, framed as a question to be answered, that a given technique must meet in order to be excluded from the scope of Directive 2001/18/EC. “Result”: Legal consequence or action to be taken depending of the fulfilment or not of the relevant criterion (i.e., depending on the answer to the relevant question under the column “Criteria”).

**Dynamic interpretation of law**

The dynamic interpretation of law is an interpretative approach which maintains that “the real meaning of a legal norm can be best disclosed at the moment of its interpretation” (Harašić, 2015: 35). It therefore pays attention to the “present societal, political, and legal context” of the legal texts under interpretation (Eskridge, 1987: 1479).

**Annex I A Part 1 Directive 2001/18/EC**

“TECHNIQUES REFERRED TO IN ARTICLE 2(2)

PART 1

Techniques of genetic modification referred to in Article 2(2)(a) are inter alia:

1. recombinant nucleic acid techniques involving the formation of new combinations of genetic material by the insertion of nucleic acid molecules produced by whatever means outside an organism, into any virus, bacterial plasmid or other vector system and their incorporation into a host organism in which they do not naturally occur but in which they are capable of continued propagation;
2. techniques involving the direct introduction into an organism of heritable material prepared outside the organism including microinjection, macroinjection, and microencapsulation;
3. cell fusion (including protoplast fusion) or hybridization techniques where live cells with new combinations of heritable genetic material are formed through the fusion of two or more cells by means of methods that do not occur naturally.”

**Annex I A Part 2 Directive 2001/18/EC**

“TECHNIQUES REFERRED TO IN ARTICLE 2(2)

[…]

PART 2

Techniques referred to in Article 2(2)(b) which are not considered to result in genetic modification, on condition that they do not involve the use of recombinant nucleic acid molecules or genetically modified organisms made by techniques/methods other than those excluded by Annex I B:

1. *in vitro* fertilization,
2. natural processes such as: conjugation, transduction, transformation,
3. polyploidy induction.”

### Leeway to Operate Out of the Scope of Directive 2001/18/EC Post Judgment

Several additional teachings deduced from the Judgment might be relevant to further clarify the situation of GMO and plant breeding in the EU, and to assess the possibility to operate out of the scope of Directive 2001/18/EC.

First, although it is indisputable that a dynamic interpretation of Annex I B Directive 2001/18/EC to include additional techniques not listed in it is not possible [see Spranger (2015) and Judgment, paras 40ff], this obstacle does not necessarily apply to the interpretation of the techniques already present in such annex nor to the requirements included or applying to the annex. Certainly, the Court does not expressly take a position on this issue (Wanner et al., 2019), but it does not close the door to a dynamic interpretation of these later aspects either. Several issues must be differentiated, particularly: flexibility as regards the definition of the techniques cited in Annex I B (i.e., what is to be understood by “mutagenesis” and “cell fusion [ … ]”); flexibility applying to the requirements from recital 17 (i.e., what is to be understood by “a number of applications” and by “a long safety record”); and flexibility in relation to the requirements stemming from the very annex (i.e., (1) “do not involve the use,” (2) “recombinant nucleic acid molecules,” and (3) “genetically modified organisms other than those produced by one or more of the techniques/methods listed” in Annex I B Directive 2001/18/EC).

With respect to the concept of “mutagenesis,” it must be noted that the Court recognizes the vagueness of Directive 2001/18/EC (see Judgment, para 43: “by referring generally to mutagenesis, that provision does not, on its own, provide any conclusive guidance as to the types of techniques/methods [ … ]”). Indeed, Directive 2001/18/EC does not address the concept of “mutagenesis” (Krämer, 2015; Spranger, 2015; Purnhagen et al. 2018b; Eriksson, 2018; Eriksson et al., 2018); but the Court implicitly admits that, at least some applications of these NBT, might eventually fit within the notion of “mutagenesis” from Directive 2001/18/EC (see the references to “new techniques/methods of mutagenesis” (Judgment, paras 48, 51, and 53) as well as the reasoning of the Court in paras 28–38).

As for the requirements stemming from recital 17 Directive 2001/18/EC in relation to the exemption of “mutagenesis” by art 3(1) in relation to Annex I B Directive 2001/18/EC, it must be remarked that if the Court had chosen to interpret them in a static way, it would rather have circumscribed its assessment to the time period prior to the approval of the Directive, but it decided instead to take into account later circumstances [see Judgment, paras 47 (“[ … ] thus far [ … ]”), 48 (“[ … ] might prove [ … ]”), 51 (“In those circumstances, [ … ]”), and 53 (“[ … ] might be [ … ]”)]. Furthermore, as remarked by Purnhagen et al. (2018a), the Court concludes by stating “that only organisms obtained by means of techniques/methods of mutagenesis which have conventionally been used in a number of applications and have a long safety record are excluded from the scope of that directive” (first conclusion, second para). Such statement is also coherent with a choice of a dynamic approach to the interpretation of Directive 2001/18/EC. If the Court had followed a static interpretation of the law, it would most likely have expressly excluded techniques used before a certain date (e.g., 2001 or 1990) or specific techniques or groups of techniques (e.g., irradiation or chemical-induced mutagenesis techniques). Furthermore, the wording used by the Court is not the result of constraints stemming from the questions referred by the *Conseil d'État* either (cf. Judgment, para 25 and conclusions). It must be deduced from the foregoing that the Judgment does not prevent the possibility of a dynamic interpretation of the techniques within Annex I B as regards the requirements stemming from recital 17.

**Static interpretation of law**

Static legal interpretation may be defined as an interpretative approach based on the idea of the “sense […] that the norm had at the time of its adoption” as its true “sense” or meaning (Harašić, 2015: 35).

Regarding the requirements contained in Annex I B Directive 2001/18/EC, the Court only mentions them by quoting the content of the annex (Judgment, para 40), and therefore no specific guidance is provided, but flexibility is neither preempted.

Second, it is clear from the aforesaid as well as from other passages of the Judgment (see, e.g., paras 53, 54 and conclusion 3), that the critical motive founding the refusal of the Court to exclude NBT from the scope of Directive 2001/18/EC is the alleged lack of “certainty” (Judgment, para 47) regarding the requirements comprised in recital 17 Directive 2001/18/EC (see paras 45, 51, 53 and conclusion 3). Therefore, if at some point those requirements imposed by recital 17 on “new techniques/methods” fitting within Annex I B Directive 2001/18/EC are proven with a reasonable degree of “certainty”, according to the teachings of the Judgment, those techniques might be excluded from the scope of Directive 2001/18/EC. However, in the light of the Judgment (see paras 50ff), even in the case of an NBT or a group of NBT eventually meeting all criteria [from recital 17 and the other applicable criteria from Directive 2001/18/EC (see **Table 1** and preceding section)], some action by the EU legislature is needed for that exclusion be feasible. We propose that a new EU directive or regulation is passed, ascertaining the fulfilment of the criteria contained in Annex I B Directive 2001/18/EC and in recital 17 Directive 2001/18/EC (listed in **Table 1**) by the relevant technique/s. This line of action is also considered by experts to be the only, or at least, the most feasible way of finding some leeway to operate with NBT out of the scope of Directive 2001/18/EC post Judgment (Purnhagen, personal com.). Such approach might be implemented within the framework of Directive 2001/18/EC as interpreted by the Judgment, without the need of a change of paradigm nor even the amendment of the scheme of Directive 2001/18/EC. Therefore, considering that a reform of Directive 2001/18/EC would be probably a lengthy process (Eriksson et al., 2018), and that such delay would have a negative impact on plant breeding (Eriksson et al., 2018), this proposal could likely work as a suitable transitory solution until a deeper reform of the EU legal regime on GMO comes. In the absence of further guidance by the CJEU on the concept of “mutagenesis” as well as of the requirements contained in Annex I B Directive 2001/18/EC, choices among the different possible interpretations (Krämer, 2015; Spranger, 2015; Jorasch, 2016; Sprink et al., 2016; Eriksson, 2018; Eriksson et al., 2018; Custers et al., 2019) will have to be made by the EU legislature. Defining in detail these choices falls completely out of the scope of the present paper; but, in the following section, the status of the most well-known breeding techniques (see **Table 2**) in the light of the aforementioned criteria from Directive 2001/18/EC interpreted according to the Judgment (see **Table 1**) is shown, further illustrating the potential reach of a limited legislative proposal like the one outlined.

**Table 2:** Plant breeding techniques comparison according to criteria defining their status under Directive 2001/18/EC in the light of the Judgment.

Breeding Technique	History of use in plant breeding	Annex I A Part 1 Directive 2001/18/EC?	Annex I A Part 2 Directive 2001/18/EC?	“[M]utagenesis” or “cell fusion [ … ]”?	“[I]nvolve [ … ] recombinant nucleic acid molecules”	“[U]se” *lato sensu* of recNA (introduction in the plant of DNA or RNA sequences)?	“[U]se” *stricto sensu of* recNA (stable insertion of “heritable” DNA sequences)?	Classification according to Directive 2001/18/EC	Out of the scope of Directive 2001/18/EC?	Potentially exemptible?
Crossing and selection (classical breeding)	Since 1870's (Mendel, 1901)	No	Yes	No	No	No	No	Art 2(2) + Annex I A Part 2	Yes	–
*In vitro* fertilization	Since 1930's (Sharma et al., 1996)	No	Yes	No	No	No	No	Art 2(2)(b) + Annex I A Part 2	Yes	–
Polyploidy induction	Since 1940's (Blakeslee and Avery, 1937)	No	Yes	No	No	No	No	Art 2(2)(b) + Annex I A Part 2	Yes	–
Random mutagenesis (chemicals, radiations)	Since 1930's (Stadler, 1928)	yes (but exempted)	No	Yes (“mutagenesis”)	No	No	No	Art 3(1) + Annex I B	Yes (exempted GMO)	–
Protoplasts fusion between sexually compatible species	Since 1970's (Carlson et al., 1972)	yes (but exempted)	No	Yes (“cell fusion [ … ]”)	No	No	No	Art 3(1) + Annex I B	Yes (exempted GMO)	–
Protoplasts fusion between sexually incompatible species	Since 1970's (Carlson et al., 1972)	yes (because not in the other annexes)	No	No	No	No	No	Art 2(2)(a) + Annex I A Part 1	No	No
“Classical” transgenesis	Since 1980's (Zambryski et al., 1983)	yes (expressly mentioned)	No	No	Yes	Yes	Yes	Art 2(2)(a) + Annex I A Part 1	No	No
Microinjection/macroinjection and microencapsulation	Since 1980's (Reich et al., 1986)	yes (expressly mentioned)	No	No	Yes^a^	Yes	Yes/No^b^	Art 2(2)(a) + Annex I A Part 1	No	No
Agro-infiltration	Since 1990's (Grimsley et al., 1986)	yes (not in the other annexes)	No	No	Yes	Yes	No	Art 2(2)(a) + Annex I A Part 1	No	No
Oligonucleotide-Directed Mutagenesis (ODM)	Since 2000's (Zhu et al., 1999)	yes (recital 17 not ascertained)	No	Yes (“mutagenesis”)	Yes/No*^c^	Yes/No^c^	No	Art 2(2)(a) + Annex I A Part 1	No	Yes*
Intragenesis	Since 2000's (Rommens et al., 2007)	yes (not in the other annexes)	No	No	Yes	Yes	Yes	Art 2(2)(a) + Annex I A Part 1	No	No
Cisgenesis	Since 2000's (Schouten et al., 2006)	yes (not in the other annexes)	No	No	No* (if no T-DNA)	No (if no T-DNA)	No (If no T-DNA)	Art 2(2)(a) + Annex I A Part 1	No	No
Transgrafting (GM scion on non-GM rootstock, or vice-versa)	Since 2000's (Lifschitz et al., 2006)	yes (not in the other annexes)	No	No	Yes	Yes/No (depends on the part harvested)	Yes/No (depends on the part harvested)	Art 2(2)(a) + Annex I A Part 1	No	No
Reverse breeding	Since 2010's (Dirks et al., 2009)	yes (not in the other annexes)	No	No	Yes	No (in the final product)	No (in the final product)	Art 2(2)(a) + Annex I A Part 1	No	No
Gene editing: Targeted mutagenesis using site-directed nucleases (SDN1) without insertion of the nuclease gene (transient transformation, RNA, RNP (ribo-nucleo protein), null segregant)	Since 2010's (Jiang et al., 2013; Woo et al., 2015)	yes (recital 17 not ascertained)	No	Yes (“mutagenesis”)	Yes/No*^c^	Yes/No^c^	Yes/No^c^	Art 2(2)(a) + Annex I A Part 1	No	Yes*
Gene editing: Targeted mutagenesis using site-directed nucleases (SDN1) with insertion of the nuclease gene	Since 2010's (Shan et al., 2013)	yes (not in the other annexes)	No	No	Yes	Yes	Yes	Art 2(2)(a) + Annex I A Part 1	No	No
Gene editing: Allele swap using site-directed nucleases (SDN2) without insertion of the nuclease gene (transient transformation, RNA, RNP (ribo-nucleo protein), null segregant)	Since 2010's (Shi et al., 2017)	yes (recital 17 not ascertained)	No	Yes (“mutagenesis”)	Yes/No*^c^	Yes/No^c^	Yes/No^c^	Art 2(2)(a) + Annex I A Part 1	No	Yes*
Gene editing: Allele swap using site-directed nucleases (SDN2) with insertion of the nuclease gene	Since 2010's (Shi et al., 2017)	yes (not in the other annexes)	No	No	Yes	Yes	Yes	Art 2(2)(a) + Annex I A Part 1	No	No
Gene editing: Targeted transgenesis using site-directed nucleases (SDN3)	Since 2010's (Li et al., 2013)	yes (not in the other annexes)	No	No	Yes	Yes	Yes	Art 2(2)(a) + Annex I A Part 1	No	No
Gene regulation using site-directed effectors (activators/repressors /epigenetic factors)	Since 2010's (Piatek et al., 2015)	yes (not in the other annexes)	No	No	Yes	Yes	Yes/No^d^	Art 2(2)(a) + Annex I A Part 1	No	No
Gene regulation using site-directed nucleases targeting RNAs (SDN4) (stable integration)	Being developed (Shmakov et al., 2015)	yes (not in the other annexes)	No	No	Yes	Yes	Yes	Art 2(2)(a) + Annex I A Part 1	No	No
RNA dependent DNA methylation (RdDM)	Being developed (Ruiz-Ferrer and Voinnet, 2009)	yes (not in the other annexes)	No	No	Yes	Yes	Yes/No^d^	Art 2(2)(a) + Annex I A Part 1	No	No
Genome editing using site-directed recombinases	Being developed in animals, soon plants (Mercer et al., 2012)	yes (not in the other annexes)	No	No	Yes	Yes	No^d^	Art 2(2)(a) + Annex I A Part 1	No	No

^a^Initially, these techniques were developed to transfer “recombinant nucleic acid molecules”.

^b^Provided that a stable insertion is not carried out.

^c^“No” provided that the allele sequence is already present in the species gene pool.

^d^“No” in case of transitory transformation or null segregant.

Nonexhaustive list of plant breeding techniques mentioned in Directive 2001/18/EC, in SAM (2017) and new approaches currently being developed, the descriptions of which can be found in the references mentioned inside the table.

All the techniques listed result in “an organism [ … ] in which the genetic material has been altered” (see question “1)” in **Table 1**).

### Analysis of the Impact of the Judgment on the Breeding Techniques

Provided that off-targeting effects and associated risks are appropriately managed, ODM, SDN1, and SDN2 are the only groups of techniques from **Table 2** with the potential to render certain applications exemptible from the scope of Directive 2001/18/EC by means of a limited legislative proposal not altering the EU GMO scheme. This conclusion has been reached through the analysis of a nonexhaustive list of plant breeding techniques mentioned in Directive 2001/18/EC, in SAM (2017) and in other sources (see **Table 2**), on the basis of the following criteria defining their status under Directive 2001/18/EC in the light of the Judgment:

- “History of use in plant breeding”: approximate time when the technique has started to be used for plant breeding (indicative publication of the first application in plants, on the basis of a search of the relevant literature on the topic). It is not meant to substitute (nor it can substitute) the assessment to be done by the EU legislature on the fulfilment of the requirements contained in recital 17 Directive 2001/18/EC. Only proposed as a proxy of the requirements stemming from recital 17 Directive 2001/18/EC for indicative purposes in the strict framework of the theoretical exercise carried out in this section. Related to questions “7)” and “8)” from **Table 1**.
- “Annex I A Part 1 Directive 2001/18/EC?”: “Does it fit in any of the techniques listed in Annex I A Part 1 Directive 2001/18/EC?” (see **Table 1**): No, yes (expressly mentioned) [expressly mentioned in Annex I A Part 1 Directive 2001/18/EC], yes (recital 17 not ascertained) [recital 17 Directive 2001/18/EC not ascertained by the EU legislature or the Judgment], yes (not in the other annexes) [it does not fit in the other annexes from Directive 2001/18/EC (see reasoning in **Table 1** and related section)], or, yes (but exempted) [it is a GMO technique according to Directive 2001/18/EC as interpreted by the Judgment, but Directive 2001/18/EC expressly exempts the technique by means of Annex I B)]. Related to question “3)” from **Table 1**.
- “Annex I A Part 2 Directive 2001/18/EC?”: “Does it fit in any of the techniques listed in Annex I A Part 2 Directive 2001/18/EC” (see **Table 1**): Yes or no. Related to question “4)” from **Table 1**.
- “‘[M]utagenesis' or ‘cell fusion [ … ]'?”: “Does it fit in the notion of “mutagenesis” or “cell fusion (including protoplast fusion) of plant cells of organisms which can exchange genetic material through traditional breeding methods”?” (see **Table 1**): No, yes (“mutagenesis”), or yes (“cell fusion [ … ]”). Related to question “5)” from **Table 1**.
- ”‘[I]nvolve [ … ] recombinant nucleic acid molecules' (recNA)”: DNA or RNA sequences containing genetic elements whose sequence and/or combination were not originally present in the species genome. (Some authors propose other definitions restricting the evaluation to the genome of the individual (not the species) or allowing to consider as nonrecombinant sequences declared as “near-identical” (Eriksson, 2018: 387) based on a threshold estimated according to the size of the genome [Eriksson, 2018)]: Yes, no, or no* (“no*” means that under a broader interpretation of “recombinant nucleic acid molecules” (e.g., when the evaluation is restricted to the genome of the individual itself), it could be a “yes”). Related to question “6)” from **Table 1**.
- “‘[U]se’ *lato sensu* of recNA”: “use of recombinant nucleic acid molecules” (*lato sensu*), i.e., introduction in the plant of DNA or RNA sequences (but not insertion of heritable recombinant DNA sequences into the genome). Related to question “6)” from **Table 1**.
- “‘[U]se' *stricto sensu* of recNA”: “use of recombinant nucleic acid molecules” (*stricto sensu*), i.e., stable insertion of heritable DNA sequences into the genome. Related to question “6)” from **Table 1**.
- “Classification according to Directive 2001/18/EC”: classification of the techniques in Directive 2001/18/EC interpreted according to the Judgment (see **Table 1** and preceding sections).
- “Out of the scope of Directive 2001/18/EC?”: status of the techniques as regards the scope of Directive 2001/18/EC interpreted according to the Judgment: yes (non-GMO), yes (exempted GMO) [exempted GMO, although according to the Judgment (conclusion 3) they may still be subjected “to the obligations laid down in that directive [Directive 2001/18/EC] or to other obligations”], or no (nonexempted GMO).
- “Potentially exemptible?”: Possibility to exclude a technique if the requirements stemming from recital 17 Directive 2001/18/EC are ascertained by the EU legislature according to the legislative proposal outlined in the present paper (based on the analysis of the Judgment, Directive 2001/18/EC and the literature (see previous sections as well as **Table 1**): No, Yes, Yes* (provided that a too broad interpretation of the requirements contained in Annex I B Directive 2001/18/EC is not adopted), or – (it does not apply because already exempted or non-GMO according to Directive 2001/18/EC interpreted according to the Judgment).

**Figure 1:**
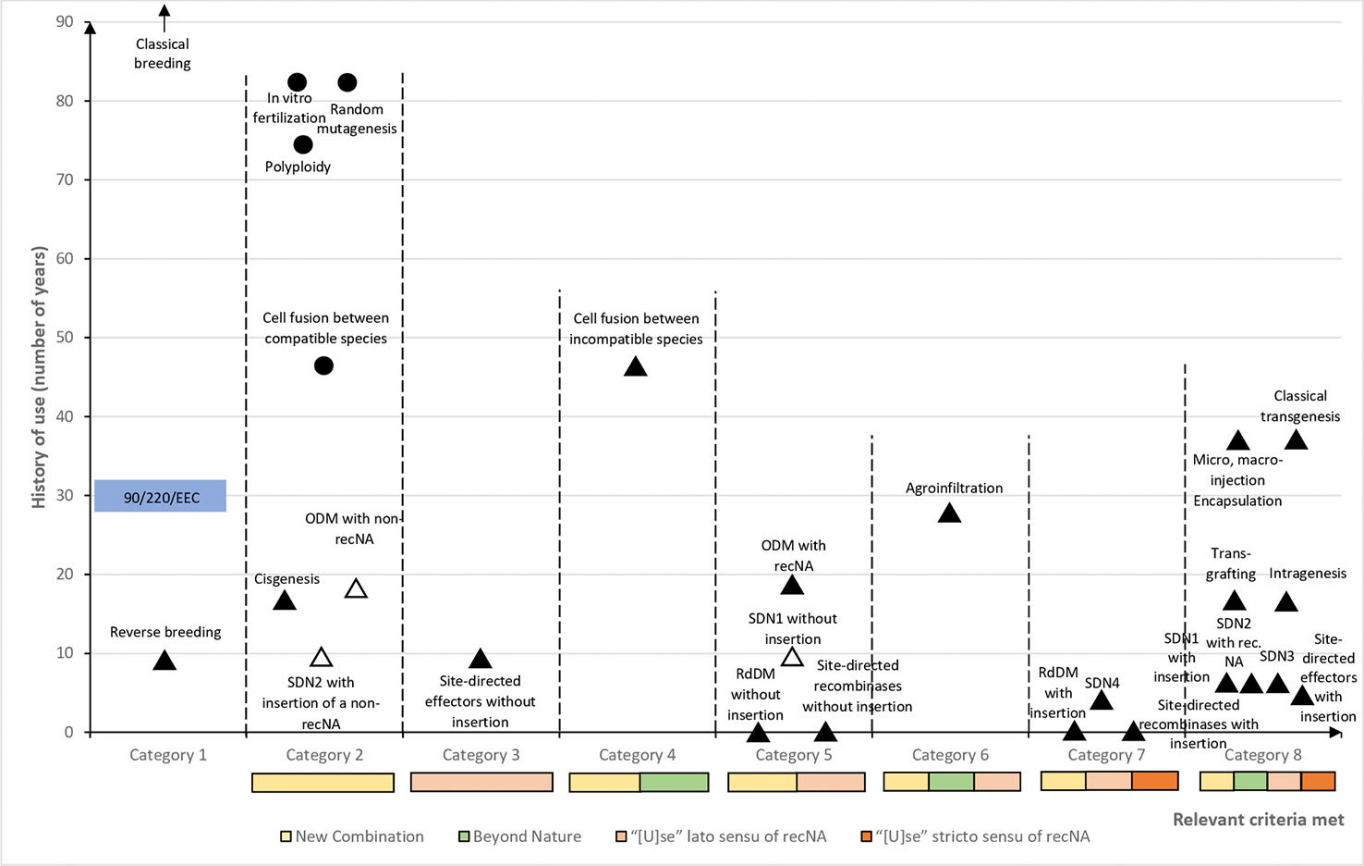
Plant breeding techniques categorized according to their history of use and criteria potentially defining their legal status. For each category of breeding techniques, criteria potentially defining their status are marked as colored bars (the sizes of which do not refer to their level of risk). Categories are arbitrarily positioned on the x-axis according to the typology and cumulation of fulfilled criteria. Each plant breeding technique is placed on the graph according to the category it belongs to, and to its history of use: black triangles and circles represent respectively techniques that are currently under the scope of Directive 2001/18/EC and exempted according to the present study, while white triangles represent techniques which could be exempted by means of a limited legislative proposal (see **Table 1** and preceding sections). Legend of the x-axis: “New Combination”: creation of a genetic variation (sequence, location) that was not present initially in the genome (see Custers et al. (2019); “Beyond nature”: genetic “[a]teration beyond what does occur naturally by mating and/or natural recombination” (Custers et al., 2019); “[U]se” *lato sensu* of recNA: “use of recombinant nucleic acid molecules” (*lato sensu*), i.e., introduction in the plant of DNA or RNA sequences, but not insertion of heritable recombinant NA sequences into the genome (related to question “6)” from **Table 1**); “[U]se” *stricto sensu* of recNA: “use of recombinant nucleic acid molecules” (*stricto sensu*), i.e., stable insertion of heritable DNA sequences into the genome (related to question “6)” from **Table 1**).

It is worth noting that the exemption or deregulation of ODM, SDN1, and SDN2 techniques (see **Table 2**) was already proposed before the Judgment [see, e.g., Eriksson (2018) and Purnhagen et al. (2018b)]. The current proposal is based on the criteria extracted from Directive 2001/18/EC as interpreted by the Judgment (see **Table 1** and preceding sections), and therefore is adapted to the current understanding of the EU legal scheme on GMO. But, in order to that exemption may work in the context of a limited legislative proposal like the one outlined, the implementation of those techniques must be limited in a way that the requirements contained in Annex I B Directive 2001/18/EC are fulfilled. In other words, only those applications of ODM, SDN1, and SDN2 that may be assimilated to mutagenesis, and that “do not involve the use of recombinant nucleic acid molecules or GMOs [ … ]” [see also Purnhagen et al. (2018b)] would be exemptible in the framework of such limited legislative proposal. It must be mentioned that, as the aforementioned techniques are based on recombinant DNA (Zhu et al., 1999; Shan et al., 2013; Shi et al., 2017), a broad interpretation of the condition “do not involve the use of recombinant nucleic acid molecules” (Annex I B Directive 2001/18/EC) would preempt the possibility of exempting any of the aforementioned techniques (see **Table 2**) without an amendment of the scheme of Directive 2001/18/EC. However, as the EU legislature did not precise what is to be understood by “not involve the use” and by “recombinant nucleic acid molecules” [see, e.g., Krämer (2015); Spranger (2015) and Eriksson (2018)], there is some margin of maneuver left to further define the condition to “not involve [ … ] recombinant nucleic acid molecules,” without being compelled to alter the scheme of Directive 2001/18/EC. But to achieve it, in addition to the requirements within Annex I B Directive 2001/18/EC, those requirements coming from recital 17 Directive 2001/18/EC (i.e., “conventionally [ … ] used in a number of applications and [ … ] a long safety record”) should be also ascertained. The recent appearance of the techniques [especially SDN1 and SDN2 (see **Table 2**)] might be perceived as a complication. However, Directive 2001/18/EC does not define how the requirements within recital 17 should be interpreted (Spranger, 2015). This means that, as long as an optimum level of “safety” is ensured, fixing the desirable threshold of those requirements is strictly a matter of legislative policy. Furthermore, it must be noted that while random mutagenesis, polyploidy and *in vitro* fertilization were rather old techniques in the nineties when the EU legal regime on GMO was born, cell fusion was just coming of age at that time (see **Table 2** and **Figure 1**). In other words, if time since the discovery and/or the popularization of the (old) breeding techniques was not an issue when the Directive was approved, it should not be a problem now with the new techniques.

In summary, the analysis of the Judgment (in the section *Interpretation of the Judgment of the CJEU of 25 July 2018*) and the reasoning and justification of the limited legislative proposal (in the section *Leeway to Operate Out of the Scope of Directive 2001/18/EC Post Judgment*) shows that such proposal, launched as a transient solution until a reform arrives, is feasible from a legal perspective. The analysis carried out in the section *Analysis of the Impact of the Judgment on the Breeding Techniques* evinces that, although fairly limited, the proposal might be also meaningful for plant breeders, providing at least some leeway to the industry.

## Conclusions

By considering targeted mutagenesis and varieties bred through these techniques as not exempted from the scope of Directive 2001/18/EC (Urnov et al., 2018), the Judgment will certainly have implications on these techniques; but, as observed by some scholars [see Martin in Science Media Centre (2018); Gelinsky and Hilbeck (2018) and Wanner et al. (2019)] as well as by CIOPORA (2018) and Jorasch (2019) from Euroseeds, it may also have an impact on traditional techniques of random mutagenesis and varieties thereof, as, from now on, varieties bred by means of traditional techniques of random mutagenesis, no matter how long they have been used, might be subjected “to the obligations laid down in that directive [Directive 2001/18/EC] or to other obligations” by EU Member States (Judgment, para 82 and conclusion 3). Although the interpretation of Directive 2001/18/EC provided by the Court is coherent with the principles governing the EU legal regime on GMO [see, e.g., Purnhagen et al. (2018a) and Eriksson (2018)], as shown by the Advocate General in its Opinion (see paras 115-117), it was not the only possible interpretation of Annex I B Directive 2001/18/EC. From now on, almost any aspect concerning those varieties (their risk assessment, labelling, cultivation, etc.) might be regulated at a national level. Even the application of Directive (EU) 2015/412 to those varieties, or stricter rules created at a national level, might eventually be dictated by Member States. Apparently though, since the Judgment came out, Member States have not regulated in that sense, and the organic sector does not seem to have urged them to proceed in that way either.

As regards NBT, the EU might consider to exempt certain techniques from the scope of Directive 2001/18/EC. In our opinion, it is clear that the Court does not dispute the classification of certain applications of NBT as a variant or species of “mutagenesis,” i.e., targeted mutagenesis; and that the reason leading to consider them as not exempted is the nonfulfilment of the requirements stemming from recital 17 Directive 2001/18/EC. Therefore, if those techniques are “used in a number of applications and [ … ] a long safety record” is ascertained, they could be exempted from the scope of Directive 2001/18/EC (provided that the specific applications of those breeding techniques fit within the concept of “mutagenesis” and comply with the requirements contained in Annex I B Directive 2001/18/EC). The analysis of the breeding techniques performed in this study shows that certain applications of ODM, SDN1, and SDN2 techniques potentially falling within the notion of mutagenesis and that “do not involve the use of recombinant nucleic acid molecules or GMOs [ … ]” could be exempted without amending the scheme of Directive 2001/18/EC. Approving a supplementary EU regulation or directive ascertaining that those techniques comply with the conditions stemming from recital 17 Directive 2001/18/EC would suffice. Certainly, even if the proposal here outlined is eventually approved, the minimum demands of the breeding sector would not be appeased by its implementation. Furthermore, it must not be forgotten that a narrow interpretation of the conditions laid down in Annex I B Directive 2001/18/EC (particularly, “do not involve the use of recombinant nucleic acid molecules”), would make the proposal unfeasible. However, if it were successfully enforced, considering that years might pass until a reform of EU legal system on GMO succeeds (Eriksson et al., 2018), and that this delay would aggravate the situation of plant breeding in the EU (Eriksson et al., 2018), such limited legislative proposal might work at least as a quick interim solution, and provide some temporary leeway to operate outside the scope of Directive 2001/18/EC.

## Data Availability Statement

All datasets generated for this study are included in the article/supplementary material.

## Author Contributions

JV-V designed the study, performed the analysis of the Judgment, participated in the analysis of the techniques, and wrote the paper. CC performed the analysis of the techniques and participated in the design and writing of the paper.

## Funding

The necessary means for the publication of this paper have been provided by the common institutional affiliation of the authors.

## Disclaimer

The information and views set out in this paper are those of the authors only and do not necessarily reflect the official opinion of the CPVO or the EU. Nothing on this paper implies a policy position of the CPVO or the EU. Neither the CPVO nor the EU nor any person acting on their behalf may be held responsible for the use which may be made of the information contained therein.

## Conflict of Interest

The authors declare that the research was conducted in the absence of any commercial or financial relationships that could be construed as a potential conflict of interest.

## Footnotes

3 Opinion of Advocate General Bobek delivered on 18 January 2018 on the case C-528/16, *Confédération paysanne and Others*, C-528/16, ECLI:EU:C:2018:20. Cited in the text as the “Opinion of Advocate General Bobek”, the “Opinion of the Advocate General”, or simply, the “Opinion”.

4 Conseil d'État, 3ème - 8ème chambres réunies, 03/10/2016, *Confederation Paysanne et autres*, No. 388649, FR:CECHR:2016:388649.20161003.

5 “Statement by the Group of Chief Scientific Advisors: A Scientific Perspective on the Regulatory Status of Products Derived from Gene Editing and the Implications for the GMO Directive.”

6 Directive (EU) 2015/412 of the European Parliament and of the Council of 11 March 2015 amending Directive 2001/18/EC as regards the possibility for the Member States to restrict or prohibit the cultivation of genetically modified organisms (GMOs) in their territory.

7 Commission Implementing Decision (EU) 2016/321 of 3 March 2016 adjusting the geographical scope of the authorisation for cultivation of genetically modified maize (Zea mays L.) MON 810 (MON-ØØ81Ø-6).

8 Judgment of 6 September 2012, *Pioneer Hi Bred Italia*, C−36/11, EU:C:2012:534.
